# Monthly Summary

**Published:** 1874-07

**Authors:** 


### MONTHLY SUMMARY.

*Neuralgia Treated by Phosphorus.*—Dr. Bradbury has in the out-patient department of Addenbrooke's Hospital, Cambridge, been lately testing the value of phosphorus in neuralgia. On the whole, he has met with considerable success from its administration. The following are two cases in which the drug effected a cure when other remedies had failed.

Case 1.—E. P., aged 24, single shop-woman, living in Cambridge, was first seen on October 20th, 1873. She had suffered from trigeminal neuralgia of the left side for three months. As the bowels were confined, and she was somewhat anæmic and the catamenia scanty. a draught containing one grain each of sulphate of iron and sulphate of quinia, five minims of dilute sulphuric acid, and half a drachm of sulphate of magnesia, in an ounce of peppermint water, was ordered thrice daily. She persevered with this mixture till December 22d, with only slight improvement. On this day the pain was very severe, and she was ordered a phosphorus capsule (= one-thirtieth of a grain of phosphorus,) to be taken twice daily after food. After taking two capsules, the pain entirely ceased; and on January 12th, 1874, the patient was discharged quite well, having had no recurrence of the neuralgia.

Case II.—E. H., aged 40, married, and living at Haslingfield, first came as out-patient on November 26th, 1873. For eight weeks she had had very severe trigeminal neuralgia, her features, being expressive of great suffering. She was suckling her baby, although the child was fifteen months old. She was directed to wean the baby, and to take a mixture containing two grains of quinia and fifteen minims of tincture of sesquichloride of iron in an ounce of water twice daily. On December 6th, there was no improvement. As cases of ague had been admitted from this locality, Dr. Bradbury, thought it possible the neuralgia might be of a malarious character, and ordered a mixture containing five minims of solution of arseniate of soda, one drachm of tincture of hop, and half an ounce of water, to be taken thrice daily, with the meals. A chloroform and belladonna liniment was also prescribed to be applied to the painful part. When next seen (December 20th,) she was as bad as ever, so a phosphorus capsule was prescribed, to be taken thrice daily after food. The patient was also ordered to rub into the temple every night a little aconitine ointment. The relief from this treatment was most marked; and when the patient was last seen, on January 31st, 1874, there had been no recurrence of the pain for more than a month.—*Brit. Med. Journal.*

---

*Chloral Poisoning Cured by Strychnia.*—A man who had taken an overdose of the hydrate of chloral, for the purpose of self-destruction, was recently taken into a hospital in Berlin. The amount of the poison was 34 grammes, or about 870 grains. He lay in a heavy sleep, respiring deeply, with congested face, and a pulse of 100. As there were no symptoms that were regarded as immediately threatening, the treatment was confined to cold applications to the head. An hour and a half later the face became livid, the pupils were widely dilated, the pulse fell to 92, the temperature rose to 103.4, and respiration became intermittent. The pneumogastrics were then faradized, and the normal respiration returned. Half an hour later symptoms of collapse set in, the pupils became contracted, the face grew pale, and the heart almost ceased to beat. A hypodermic injection of one twenty-fifth of a grain of strychnia was then given. The beating of the heart was materially strengthened, and the pupils were dilated. These favorable symptoms, however, lasted but a short time, and the face again became llvid, the heart-beats were fainter, and the pupils did not re-act. At this time about one twenty-fifth of a grain of strychnia was administered hypodermically. The action of the heart was again restored, but the respiration was labored. It was necessary to resort to faradization, which was kept up at intervals during the night and part of the next day. On the same afternoon the patient had recovered his consciousness. Notwithstanding the large quantity of chloral taken, there were no gastric symptoms, which was explained on the ground that the patient had eaten largely of rye bread before taking the poison. This case is illustrated of the value of strychnia in poisoning by chloral. Liebreich was the first to show the physiological antagonism between the two substances. It is noteworthy that faradization played an important but secondary role in the case.—*Berl. Klin. Woch.*, 1, 1874.

---

*Blue Pus.*—It is well known that pus, and the dressing of neglected wounds, sometimes show a blue color. The *American Journal of the Medical Science* contains an article on this subject condensed from the *Medical Times and Gazette*, and one from the *Archives Gen. de Med.* This blue color of pus may become epidemic, as was the case in M. Gosselin's wards in the Charite at Paris. Cases are also on record in which the normal secretions, as sweat, milk, and urine, have been of this color. Two sources for the color are indicated: one, the hæmoglobin; the other, the indican of the urine. Hæmoglobin, effused, assumes the varying colors seen in a bruise; in an old clot it becomes hæmatoidin, identical with the red coloring matter of the bile. The action of nitric acid, which is a process of oxidation, produces a blue tint in the bile. Perhaps a similar oxidation of hæmoglobin gives rise to the blue of the secretion, or the pus,

Various theories have been advanced to explain the blue coloring of the dressing wounds. One, favored by Lucke, is that it is due to vibrios. [Our readers will bear in mind the theory that all decomposition is caused by fungi, or other microscopic organisms.] This theory of the vibrios would explain the epidemics, if we might so call them, which occur where there is a neglect of cleanliness, in a hot, moist weather.

A blue coloring matter, called pyocyanine, has been isolated from blue pus, which resembles indican, a blue coloring matter occurring as a normal constituent of urine and probably of the blood also. This indican may be the source of the blue color of the pus and secretion.

*The Archives de Med.* considers the blue color to be of three kinds: 1st. The coloration resulting from the modification of certain humors—or true blue coloration; 2d. Coloration due to fungi—false blue coloration; 3d. Coloration due to a substance still unknown—false blue suppuration. The name *cyanchorse* is proposed for the last. It commences and ceases suddenly, nct only where there is a wound, but where the parts are sound. Its duration is variable It produces no modification in the local condition of the wound, or the general health of the patient. Its progress is similar to that of erysipelas. It is sometimes epidemic. It occurs most frequently when the atmosphere is moist and warm and contains ozone, and during storms. Its presence is a favorable prognostic.—*Med. Examiner.—Missouri Clinical Record.*

---

*How to Check Coughs.*—Dr. Brown-Sequard, in his late Boston Lectures, says that there are many facts which show that morbid phenomena of respiration can always be stopped by the influence of arrest. Coughing, for instance, can be stopped by pressing on the nerve of the lip in the neighborhood of the nose. A pressure there may prevent a cough when it is beginning. Sneezing may be stopped by the same mechanism. Pressing in the neighborhood of the ear, right in front of the ear, may stop coughing. It is also preventive of hiccough, but much less so than of sneezing or coughing. Pressing very hard on the top of the mouth inside is also a means of stopping coughing. And he adds, that the will has immense power there. There was a French nurse who used to say, "The first patient who coughs here will be deprived of his food to day." It was exceedingly rare that a patient coughed then.—*Med. and Surg. Reporter.*

---

*Determination of the Age of the Human Embryo by the Evolution of the Teeth.*—M. E. Magitot has been occupied in the solution of this problem, so important from a physiological point of view. He has determined that a maxilla or even a fragment of a maxilla may now suffice to fix the age of the embryo. The author gives in a table his conclusions, which we regret to be unable to publish for want of space. In a communication about to be published, the author intends to use the evolution of the dental follicles in determination of the age of the newly-born child.—*La France Medicale*, May 6, '74.—*The Clinic.*

---

*Oxygen Gas as a Remedy in Disease.*—Prof. J. L. Cabell, M. D., Univ., Va., regards oxygen gas as a most valuable agent in all diseases involving defective respiration, and as a tonic to improve assimilation in chronic diseases.—*Va. Med. Monthly.*

